# Psychometric Properties of the MOLDES Questionnaire in a Clinical Sample of Patients With Psychotic Spectrum Disorders

**DOI:** 10.62641/aep.v54i3.2176

**Published:** 2026-06-15

**Authors:** Juan Jesús Muñoz García, Ricardo M. Hodann-Caudevilla, Alfonso García Castaño, Sergio Aguilera Garrido, Rafael Durán Tischhauser, Álvaro Pico Rada, Rafael Salom

**Affiliations:** ^1^Centro de San Juan de Dios, Orden Hospitalaria San Juan de Dios, 28350 Ciempozuelos, Madrid, Spain; ^2^Fundación Hospitalarias Zaragoza, Hermanas Hospitalarias, 50190 Zaragoza, Spain; ^3^Hospital Universitario Infanta Sofía, 28702 San Sebastián de los Reyes, Madrid, Spain; ^4^Hospital Universitario Infanta Cristina, 28981 Parla, Madrid, Spain; ^5^Centro de Salud Mental Santa Mónica, 28522 Rivas-Vaciamadrid, Madrid, Spain; ^6^Clínica Nuestra Señora de la Paz, Orden Hospitalaria San Juan de Dios, 28033 Madrid, Spain; ^7^Department of Psychology, Universidad Europea de Valencia, 46010 Valencia, Spain

**Keywords:** psychotic disorders, schizophrenia, adaptation, psychological, psychometrics, inpatients

## Abstract

**Background:**

Psychotic spectrum disorders are associated with persistent impairments in psychosocial functioning that are not fully explained by symptom severity, particularly in individuals with chronic courses and prolonged hospitalization. Beyond psychopathological symptoms, relatively stable cognitive-emotional styles may influence long-term adaptation, subjective recovery, and quality of life in severe mental disorders. To examine the psychometric properties of the MOLDES questionnaire in a clinical sample of patients with psychotic spectrum disorders receiving long-term psychiatric care.

**Methods:**

A cross-sectional observational study was conducted in a sample of 83 clinically stable male patients with psychotic spectrum disorders hospitalized in a long-term psychiatric care unit. Internal consistency of the global MOLDES score and its three dimensions (Vital Spontaneity, Adjustment, and Optimization) was assessed using Cronbach’s alpha. Convergent and discriminant validity were explored through Spearman’s correlations with psychotic symptom severity, depressive symptoms, internalized stigma, and quality of life.

**Results:**

The global MOLDES score and its dimensions showed acceptable internal consistency, with Cronbach’s alpha values ranging from 0.70 to 0.77. No significant associations were observed between MOLDES dimensions and psychotic or depressive symptom severity, supporting discriminant validity. In contrast, Vital Spontaneity and Adjustment were negatively correlated with internalized stigma, while the Optimization dimension showed a positive association with quality of life.

**Conclusions:**

These findings provide preliminary evidence supporting the reliability and validity of the MOLDES questionnaire in patients with chronic psychotic disorders. The instrument appears to capture adaptive cognitive-emotional styles that are largely independent of symptom severity and meaningfully related to relevant psychosocial outcomes, suggesting its potential usefulness as a complementary assessment tool in long-term psychiatric care and rehabilitation-oriented settings.

## Introduction

Psychotic spectrum disorders are severe mental conditions characterized not only by the presence of positive and negative symptoms, but also by persistent impairments in psychosocial functioning, autonomy, and quality of life. Even during clinically stable phases, many individuals with schizophrenia and related disorders experience long-term difficulties in social participation, emotional regulation, and adaptive functioning, which are not fully explained by symptom severity alone [1, 2]. In recent years, growing attention has been given to moving beyond symptom-based models and incorporating broader functional and psychosocial dimensions into assessment and intervention in schizophrenia, including factors such as stigma, sleep-related cognition, and social functioning [3, 4, 5]. Research in long-term psychiatric settings has emphasized psychosocial functioning and subjective experience as key indicators of clinical complexity and recovery potential in severe mental disorders, underscoring the need for assessment tools that capture how individuals habitually engage with their lived reality [6, 7, 8].

Beyond core psychopathological symptoms, people with psychotic spectrum disorders tend to develop enduring patterns in the way they process experiences, regulate emotions, and respond to environmental demands. These habitual cognitive-emotional tendencies influence everyday functioning, interpersonal engagement, and subjective recovery, and may remain relatively stable despite changes in symptom intensity [9, 10, 11]. From this perspective, cognitive-emotional styles can be understood as a transdiagnostic layer of functioning that contributes to explaining heterogeneity in psychosocial adjustment among individuals with similar clinical diagnoses. Prior research has shown that these styles are closely linked to important psychosocial outcomes in psychosis, including self-concept, internalized stigma, emotional well-being, and quality of life, in some cases exerting a stronger influence on daily functioning than positive or negative symptoms themselves [12, 13]. For this reason, assessing habitual cognitive-emotional patterns are particularly relevant in chronic and institutionalized populations, where long-term adaptation, resilience, and subjective experience become central therapeutic targets [4, 7, 8].

Although the clinical relevance of cognitive-emotional styles and adaptive functioning in psychosis is increasingly acknowledged, their systematic assessment continues to pose methodological difficulties in both research and routine practice [14]. Most instruments commonly used in schizophrenia focus on symptom severity, neurocognitive deficits, or externally defined functional outcomes, often neglecting the subjective and habitual ways in which individuals relate to their inner experiences and everyday reality [15, 16]. While measures of insight, stigma, or quality of life provide valuable complementary perspectives, they usually capture circumscribed domains rather than offering an integrated picture of cognitive-emotional style [15, 17, 18]. Furthermore, many available tools were not specifically designed to assess relatively stable patterns of emotional regulation, motivational orientation, and experiential openness, which may play a critical role in long-term psychosocial adaptation, particularly in chronic and institutionalized populations [4, 6, 19]. This situation highlights the need for assessment instruments capable of capturing global cognitive-emotional styles of adaptation, with adequate psychometric soundness and clinical relevance.

Within this context, the MOLDES questionnaire (Test de Estrategias Cognitivo-emocionales, translated as Cognitive-Emotional Coping Modes and Strategies) [20] was developed as a comprehensive self-report measure aimed at assessing habitual cognitive-emotional styles in the way individuals relate to and manage reality. Rather than focusing on symptoms, MOLDES conceptualizes adaptation as stable cognitive-emotional styles reflected in emotional expressiveness, regulatory capacity, and growth orientation, organized into three higher-order dimensions: Vital Spontaneity, Adjustment, and Optimization. This dimensional and non-pathologizing approach allows for the assessment of global cognitive-emotional styles from a psychosocial perspective, capturing aspects of resilience, flexibility, and experiential openness that are often overlooked by symptom-centered instruments. Previous research has supported the psychometric robustness of the MOLDES in non-psychotic and mixed clinical populations, showing high internal consistency and meaningful associations with indicators of psychological adjustment and well-being [21]. However, despite its potential clinical relevance, the instrument has not yet been systematically validated in samples of individuals with psychotic spectrum disorders, particularly in chronic and long-term care settings. Given the distinctive clinical characteristics of these populations, including prolonged institutionalization, functional disability, and complex psychosocial needs, examining the psychometric properties of the MOLDES in this context represents a necessary step to determine its applicability and usefulness as a complementary assessment tool in severe mental disorders.

The primary objective of the present study was to examine the psychometric properties of the MOLDES questionnaire in a clinical sample of patients with psychotic spectrum disorders receiving long-term psychiatric care. Specifically, the study aimed to assess the internal consistency of the global MOLDES score and its three higher-order dimensions (Vital Spontaneity, Adjustment, and Optimization). In addition, convergent and discriminant validity were explored by analyzing the associations between MOLDES dimensions and a set of relevant clinical and psychosocial variables, including psychotic symptom severity, depressive symptoms, internalized stigma, and quality of life. Through this approach, the study sought to determine the potential applicability and clinical usefulness of the MOLDES as a complementary assessment tool for understanding adaptive cognitive-emotional functioning in individuals with severe and chronic psychotic disorders.

## Materials and Methods

### Study Design

The sample consisted of 83 male patients diagnosed with psychotic spectrum disorders, primarily paranoid and residual schizophrenia, as well as schizoaffective disorder. Diagnoses were established through a structured diagnostic interview in accordance with international diagnostic criteria. All participants were recruited between February 2023 and December 2024 from the Centro de San Juan de Dios de Ciempozuelos (Ciempozuelos, Madrid, Spain), where they were continuously hospitalized and receiving their usual psychiatric treatment. Participants were recruited using a consecutive sampling strategy among all patients meeting eligibility criteria during the recruitment period.

Only patients in a clinically stable phase were included. Clinical stability was defined as the absence of acute symptom exacerbations requiring clinical intervention and no relevant changes in psychopharmacological treatment during at least the preceding month, as confirmed by the treating psychiatrists. Individuals with comorbid intellectual disability were excluded.

Inclusion criteria comprised a confirmed diagnosis of a psychotic spectrum disorder according to the International Classification of Diseases, 10th Edition (ICD-10), current clinical stability (no acute exacerbations in the previous month), and no significant modifications in pharmacological treatment during that period. In addition, participants were required to demonstrate sufficient cognitive and functional capacity to understand the study procedures and to complete the assessment instruments independently or with minimal assistance (no additional assistance beyond the standard instructions provided at the beginning of the evaluation was required). Furthermore, exclusion criteria included comorbid intellectual disability, the presence of acute symptoms likely to interfere with comprehension of instructions or sustained participation in the assessment, and inability to complete the questionnaires. The sample was composed exclusively of men due to the low proportion of female patients admitted to the unit, together with the strict inclusion criteria related to clinical stability and response capacity, which further limited their potential participation.

### Materials

A battery of questionnaires and clinical interviews was administered to collect sociodemographic, clinical, and psychosocial information, including global functioning, internalized stigma, depressive symptoms, and quality of life. Data collection was conducted through individual assessment sessions in a clinically stable context and in close coordination with the clinical staff of the unit.

#### Sociodemographic Data

Sociodemographic information was collected for all participants, including age, gender, marital status, educational level, current occupational status, as well as information regarding civil incapacitation and officially recognized degree of disability. Relevant clinical history variables were obtained from clinical records and interviews.

#### Clinical and Psychosocial Measures

MOLDES (Test de Estrategias Cognitivo-emocionales; Cognitive-Emotional Coping Modes and Strategies) [20]: The MOLDES is an 87-item self-report instrument developed to assess habitual cognitive-emotional styles through which individuals interpret and manage their lived reality. The questionnaire is structured around three higher-order dimensions (Vital Spontaneity, Adjustment, and Optimization), which together reflect different aspects of adaptive cognitive-emotional functioning. Vital Spontaneity reflects emotional expressiveness, enthusiasm, and openness to experience; Adjustment refers to emotional and behavioral regulation in response to situational demands; and Optimization captures a growth-oriented tendency toward personal development, well-being, and the effective use of adaptive resources over time. Items are rated on a 5-point Likert scale ranging from 1 (totally disagree) to 5 (totally agree). Raw scores were calculated for each dimension and subsequently transformed to a standardized 0–100 scale to facilitate interpretation and comparison across dimensions. This approach is consistent with the use of standardized metrics and normative interpretation described in the MOLDES manual [20]. Higher scores indicating greater expression of the respective cognitive-emotional style. The original version of the questionnaire showed excellent internal consistency (α = 0.90).

Positive and Negative Syndrome Scale (PANSS) [22]: The PANSS is a clinician-administered, semi-structured interview specifically designed to assess symptom severity in schizophrenia. It consists of 30 items evaluating positive symptoms, symptom balance (positive-negative symptoms), and general psychopathology, including an item assessing insight into illness and need for treatment. Items are rated on a 7-point scale ranging from 1 (absent) to 7 (extreme), yielding total scores between 30 and 210. The PANSS Composite score provides an index of symptom balance by subtracting negative from positive symptom scores. The Spanish version has demonstrated high interrater reliability, with intraclass correlation coefficients ranging from 0.84 to 0.93.

Estigma Interiorizado de Enfermedad Mental (EIEM; the Spanish version of the Internalized Stigma of Mental Illness Inventory) [23]: This instrument was used to assess the subjective experience of internalized stigma. The questionnaire comprises 29 items covering dimensions such as alienation, stereotype endorsement, perceived discrimination, and social withdrawal. Items are rated on a 4-point Likert scale (1 = strongly disagree to 4 = strongly agree). Although the original scoring procedure recommends calculating the mean score across items (range 1–4), in the present study the total score was computed as the sum of all item responses (possible range: 29–116) to maintain consistency with the scoring approach used for the other instruments included in the analyses. Higher scores indicate greater internalized stigma. The Spanish adaptation has demonstrated excellent internal consistency and test–retest reliability, with coefficients of 0.91 and 0.95, respectively.

WHO Quality of Life-BREF (WHOQOL-BREF) [24]: WHOQOL-BREF is a 26-item self-report instrument assessing quality of life across four domains: Physical Health, Psychological Health, Social Relationships, and Environment. Items are rated on a 5-point Likert scale (1 = very poor/very dissatisfied to 5 = very good/very satisfied). Although the WHOQOL-BREF is commonly scored using domain-specific transformations to a 0–100 scale, in the present study a global quality-of-life index was calculated by summing the raw scores of all 26 items (possible range: 26–130), with higher scores indicating better perceived quality of life. A global summed score was used to reflect overall perceived quality of life. The Spanish version has demonstrated adequate to good internal consistency across domains, with Cronbach’s alpha values ranging from 0.69 to 0.90.

Calgary Depression Scale for Schizophrenia (CDSS) [25]: The CDSS is a clinician-administered instrument specifically developed to assess depressive symptoms in individuals with schizophrenia, while minimizing overlap with positive, negative, and extrapyramidal symptoms. It consists of 9 items, each rated from 0 (absent) to 3 (severe), yielding a total score between 0 and 27. The scale has shown good internal consistency, with Cronbach’s alpha values ranging from 0.70 to 0.90.

### Ethical Considerations

All study procedures were reviewed and approved by the Ethics and Research Committee of Hospital Universitario 12 de Octubre (approval code: 18/101; approval date: 10 April 2018). The study was conducted at Centro San Juan de Dios (Ciempozuelos), which does not have its own institutional research ethics committee. Therefore, ethical review and approval were obtained through the Ethics Committee of Hospital Universitario 12 de Octubre, an accredited committee authorized to evaluate research projects conducted in collaborating clinical centers. All procedures were carried out in accordance with the Declaration of Helsinki and current ethical standards for research involving human participants. Clinical psychologists from the unit verified patient eligibility, explained the study objectives, and addressed any questions prior to obtaining written informed consent. Participation was entirely voluntary, and participants were informed of their right to withdraw at any time without any consequences for their clinical care. No financial compensation was provided for participation.

### Statistical Analysis

Reliability analyses were conducted using the statistical software Jamovi (version 2.6; Jamovi project, Sydney, Australia) [26]. First, the assumptions of normality and linearity were examined. While most variables approximated a normal distribution, depressive symptoms showed a significant deviation from normality (Shapiro–Wilk *p* < 0.001). For consistency across instruments, total and dimensional scores were computed using summed raw item scores rather than item means. As summed and mean scores represent linear transformations of one another, this scoring approach does not affect reliability coefficients or correlational analyses but facilitates comparability across measures.

Then, the internal consistency of the MOLDES questionnaire was evaluated for both the total scale and its dimensions using Cronbach’s alpha (α). Also, to examine the convergent and discriminant validity of the MOLDES, a matrix of Spearman’s correlation coefficients was computed between the main dimensions of the questionnaire (Vital Spontaneity, Adjustment, and Optimization) and several clinical and functional indicators. Given the non-normal distribution of depressive symptoms, a non-parametric approach was adopted. Specifically, associations were analyzed with the total scores of the positive symptoms, Symptom Balance, insight, depression symptoms, internalized stigma, and quality-of-life score. The significance level was set at *p* < 0.05 (two-tailed) for all analyses.

## Results

The sample consisted of 83 male patients with chronic psychotic spectrum disorders receiving long-term inpatient care. The mean age was 47.22 years (SD = 9.75). The mean age at first psychiatric hospitalization was 21.12 years (SD = 4.81). Participants had experienced an average of 10.85 psychiatric admissions (SD = 10.62), and the mean length of stay in long-term care units was 8.25 years (SD = 9.01), reflecting substantial chronicity and clinical complexity. Table 1 presents descriptive statistics for the main psychological and clinical variables assessed in the sample, including measures of central tendency, variability, and range, providing an overall characterization of the participants.

**Table 1.** **Descriptive statistics**.

Variables	Mean	Standard deviation	Median (IQR)	Min.	Max.	Shapiro–Wilk	
						W	*p*
Vital spontaneity	58.21	11.20	-	34	86	0.99	0.74
Adjustment	61.31	10.39	-	27	96	0.97	0.02
Optimization	68.98	12.43	-	39	96	0.99	0.48
Insight	3.46	0.80	-	2	5	0.97	0.04
Depression symptoms	-	-	2 (0.00–8.00)	0	21	0.80	0.001
Symptom balance	–2.90	7.09	-	–19	10	0.98	0.45
Positive symptoms	22.16	6.28	-	8	36	0.98	0.26
Internalized stigma	65	13.4	-	32	97	0.99	0.64
Quality of life	89.51	14.32	-	56	130	0.99	0.55

Note: Non-normally distributed variables are presented as median and interquartile range (IQR), whereas normally distributed variables are presented as mean and standard deviation. Vital spontaneity, adjustment, and optimization correspond to the three higher-order dimensions of MOLDES. Positive Symptoms were derived from the Positive and Negative Syndrome Scale (PANSS) Positive subscale. Symptom Balance represents the difference between the PANSS Positive and Negative subscales (Positive – Negative). Insight corresponds to the PANSS General Psychopathology subscale. Depressive symptoms were assessed using the Calgary Depression Scale for Schizophrenia. Internalized stigma was measured using the total Estigma Interiorizado de Enfermedad Mental score. Quality of life was assessed using the total WHO Quality of Life-BREF score.

### Reliability Analysis: Global Scale

The internal consistency of the global MOLDES score was acceptable (α = 0.747) and did not improve following the removal of any individual item. Item-level analyses indicated that alpha and omega coefficients remained stable across item deletions, suggesting adequate homogeneity of the scale while retaining sufficient item-specific variance.

### Reliability Analysis: Dimensional Scores

Internal consistency was examined separately for each MOLDES dimension. Overall, the dimensions showed acceptable to good internal consistency in the clinical sample, with alpha values ranging from 0.70 to 0.77. The Vital Spontaneity dimension demonstrated acceptable internal consistency (α = 0.73). Item-deletion analyses indicated that the removal of any item within this dimension did not result in a meaningful increase in the alpha coefficient. Similarly, the Adjustment dimension showed reliability values ranging approximately between α = 0.70 and 0.74, with no substantial improvements observed following the deletion of individual items. The Optimization dimension yielded the highest internal consistency estimates, reaching a Cronbach’s alpha of α = 0.77. As with the other dimensions, item removal did not lead to significant increases in the alpha coefficient.

### Convergent and Discriminant Validity

#### Intercorrelations Among MOLDES Dimensions

First, significant correlations were observed among the MOLDES dimensions themselves (Table 2). Vital Spontaneity was positively associated with Adjustment (*r* = 0.460, *p* < 0.001), whereas both dimensions showed significant negative correlations with Optimization (*r* = –0.341, *p* = 0.002 and *r* = –0.262, *p* = 0.017, respectively).

**Table 2.** **Correlation analysis of key variables: vital spontaneity, adjustment, optimization, insight, depression symptoms, positive symptoms and symptom balance, internalized stigma, and quality of life**.

Variable	1	2	3	4	5	6	7	8	9
1. Vital spontaneity	-								
2. Adjustment	0.460***	-							
3. Optimization	–0.341**	–0.262*	-						
4. Insight	0.100	–0.021	–0.116	-					
5. Depression symptoms	0.062	0.026	–0.097	–0.068	-				
6. Symptom balance	–0.018	–0.081	0.068	–0.100	0.053	-			
7. Positive symptoms	–0.045	0.006	–0.053	0.032	–0.136	0.243*	-		
8. Internalized stigma	–0.218*	–0.317**	–0.066	–0.005	–0.271*	0.019	0.275*	-	
9. Quality of life	0.141	0.130	0.325**	–0.097	–0.086	–0.086	–0.136	0.132	-

Note: ***Significant correlation at 0.001 level; **Significant correlation at 0.01 level; *Significant correlation at 0.05 level. Vital spontaneity, adjustment, and optimization correspond to the three higher-order dimensions of MOLDES. Positive Symptoms were derived from the Positive and Negative Syndrome Scale (PANSS) Positive subscale. Symptom Balance represents the difference between the PANSS Positive and Negative subscales (Positive − Negative). Insight corresponds to the PANSS General Psychopathology subscale. Depressive symptoms were assessed using the Calgary Depression Scale for Schizophrenia. Internalized stigma was measured using the total Estigma Interiorizado de Enfermedad Mental score. Quality of life was assessed using the total WHO Quality of Life-BREF score.

#### Associations With Symptom Measures (Discriminant Validity)

With regard to clinical indicators, no significant correlations were found between the MOLDES dimensions and overall psychotic symptom severity, including positive symptoms and symptom balance. Likewise, no significant associations were observed between the MOLDES dimensions and depressive symptom severity.

#### Associations With Psychosocial Variables (Convergent Validity)

In contrast, significant associations were found with relevant psychosocial variables. The Optimization dimension was positively correlated with overall quality of life (*r* = 0.325, *p* = 0.003). In addition, Vital Spontaneity and Adjustment showed moderate significant negative correlations with internalized stigma, with coefficients of *r* = –0.275 (*p* = 0.012) and *r* = –0.317 (*p* = 0.003), respectively.

## Discussion

The aim of the present study was to evaluate the psychometric properties of the MOLDES questionnaire in a clinical sample of patients with psychotic spectrum disorders receiving long-term psychiatric care. Overall, the findings suggest that the MOLDES provides clinically relevant information beyond conventional symptom-focused assessments in long-term psychiatric care.

### Psychometric Performance of the MOLDES in a Chronic Psychosis Sample

With regard to internal consistency, the global MOLDES score and its three higher-order dimensions showed acceptable reliability indices, with Cronbach’s alpha values comparable to those reported in non-psychotic and mixed clinical samples. Although reliability coefficients were somewhat lower than those described in the original validation study, they remain within acceptable ranges for research purposes, particularly considering the clinical complexity, chronicity, and cognitive heterogeneity of the present sample. Importantly, item-level analyses indicated that the removal of individual items did not result in meaningful improvements in internal consistency, suggesting adequate homogeneity of the scale while preserving sufficient content breadth.

These results support the feasibility of using the MOLDES questionnaire as a complementary assessment tool in chronic psychosis, provided that its scores are interpreted within a broader clinical framework and not as a direct proxy for symptom severity or clinical status.

### Discriminant Validity: Independence from Symptom Severity

A central finding of the study was the absence of statistically significant associations between MOLDES dimensions and psychotic symptom severity, including positive, balance, and general symptoms, as well as depressive symptoms. Although this pattern is theoretically consistent with the conceptualization of cognitive-emotional styles as relatively stable patterns of adaptation rather than reflections of transient psychopathological states, it should be interpreted with caution. Given the modest sample size, the study may have been underpowered to detect small-to-moderate correlations. Therefore, the absence of significant associations cannot be considered definitive evidence of discriminant validity, but rather preliminary support that warrants replication in larger samples.

This result is in line with previous research indicating that functional outcomes, subjective recovery, and psychosocial adjustment in psychosis are only partially explained by symptom burden [1, 2]. In chronic and institutionalized populations, long-term adaptation appears to depend more on habitual patterns of emotional regulation and management of environmental demands than on fluctuations in psychotic symptoms [7]. Accordingly, the absence of associations between MOLDES scores and symptom measures should not be interpreted as a limitation, but rather as evidence that the instrument captures a distinct and clinically meaningful level of psychosocial functioning.

This distinction may be particularly relevant in long-term psychiatric care settings, where symptom stability is often achieved while substantial heterogeneity persists in psychosocial functioning, subjective well-being, and recovery trajectories. In this context, instruments such as the MOLDES may help identify clinically relevant differences that are not adequately reflected by conventional symptom-based assessments.

### Convergent Validity: Associations with Stigma and Quality of Life

In contrast to symptom measures, significant associations were observed between MOLDES dimensions and key psychosocial variables. Specifically, Vital Spontaneity and Adjustment were negatively associated with internalized stigma, while Optimization showed a positive association with quality of life. These findings are theoretically coherent and clinically relevant. Although moderate in size, these effects are consistent with prior psychosocial research in chronic psychosis, where multiple interacting determinants limit the magnitude of individual associations [8, 12].

Internalized stigma has been consistently identified as a major determinant of reduced self-esteem, social withdrawal, and poorer recovery outcomes in individuals with psychotic disorders [12, 13]. The present results suggest that more adaptive cognitive-emotional styles, characterized by emotional expressiveness, regulatory capacity, and flexible engagement with reality, may be associated with lower levels of internalized stigma. This interpretation is consistent with recent evidence highlighting the role of psychosocial and experiential processes in mediating the impact of stigma on subjective well-being and adaptation in chronic psychotic disorders [27].

Similarly, the positive association between the Optimization dimension and quality of life supports the notion that a growth-oriented and future-directed cognitive-emotional style may contribute to better subjective well-being, even in the context of severe and persistent mental illness. This finding aligns with recovery-oriented perspectives that emphasize adaptive functioning, meaning-making, and personal development beyond symptom remission, particularly in long-term psychiatric care settings [6].

A further aspect worth noting is the negative association observed between Optimization and the dimensions of Vital Spontaneity and Adjustment. Although all three are conceptualized as adaptive, they represent differentiated cognitive-emotional styles rather than components of a single unidimensional construct. Optimization reflects a structured, goal-directed orientation toward growth, which may not necessarily co-occur with higher spontaneous expressiveness or contextual flexibility, particularly in chronically institutionalized populations [10, 11]. This pattern therefore likely reflects differentiated adaptive profiles rather than scoring artifacts.

### Clinical Implications in Long-Term Psychiatric Care

Overall, the results suggest that the MOLDES questionnaire can contribute clinically relevant information that goes beyond what is captured by conventional symptom-focused assessments in long-term psychiatric care settings. In populations characterized by prolonged institutionalization and complex psychosocial needs, evaluating habitual cognitive-emotional styles may help clinicians better understand individual differences in adaptation, resilience, and recovery potential.

By emphasizing enduring patterns of emotional regulation, engagement with reality, and adaptive psychosocial functioning, the MOLDES offers a conceptual framework that is consistent with rehabilitation-oriented models of care [6]. From a practical standpoint, the instrument may assist in identifying adaptive strengths and vulnerabilities, informing individualized psychosocial interventions, and enriching clinical formulations beyond symptom severity alone.

### Limitations and Future Directions

Several limitations should be acknowledged. First, the sample was composed exclusively of male patients from a single long-term psychiatric care unit, which substantially limits the generalizability of the findings. Psychotic spectrum disorders show well-documented gender differences in clinical course, cognitive-emotional functioning, internalized stigma, and quality of life, with some studies reporting higher levels of internalized stigma and affective burden among female patients. Consequently, the psychometric performance, factor structure, and correlational patterns of the MOLDES dimensions may differ in gender-diverse samples.

In addition, the exclusive inclusion of chronically institutionalized individuals may introduce contextual bias, as prolonged hospitalization, high levels of disability, and reduced environmental variability can shape cognitive-emotional styles and response patterns to self-report instruments. The present findings should therefore be interpreted as preliminary and context-specific, reflecting a male, long-term inpatient population with chronic psychosis. Replication in gender-diverse samples and in mixed clinical settings, including outpatient and community-based populations, represents a critical next step to determine the broader applicability and stability of the MOLDES across contexts and illness stages.

Second, the relatively modest sample size represents an important methodological limitation. Psychometric research, particularly studies examining construct validity through correlational analyses, typically benefits from larger samples to ensure adequate statistical power and stable parameter estimation. The present study may have been underpowered to detect small-to-moderate associations between MOLDES dimensions and symptom severity, which limits the strength of conclusions regarding discriminant validity. In addition, the sample size precluded meaningful subgroup analyses across different psychotic spectrum diagnoses (e.g., paranoid schizophrenia versus schizoaffective disorder), preventing exploration of potential diagnostic heterogeneity in MOLDES performance.

Future studies should aim to replicate these findings in larger and more diverse samples, including female patients and individuals at earlier stages of illness. Longitudinal designs would be particularly valuable to examine the stability of cognitive-emotional styles and their predictive value for functional outcomes, relapse, and recovery trajectories. Further research could also explore the responsiveness of the MOLDES to psychosocial and rehabilitative interventions, clarifying its potential role as an outcome measure in both clinical research and routine practice.

## Conclusions

The present study provides preliminary evidence supporting the reliability and validity of the MOLDES questionnaire in a clinical sample of patients with psychotic spectrum disorders receiving long-term psychiatric care. The findings suggest that MOLDES captures adaptive cognitive-emotional styles that are largely independent of symptom severity and meaningfully associated with relevant psychosocial outcomes, such as internalized stigma and quality of life. Taken together, these results highlight the potential utility of the instrument as a complementary assessment tool for understanding psychosocial adaptation and recovery-related processes in chronic psychosis, and support the inclusion of cognitive-emotional style assessment within broader, rehabilitation-oriented evaluation frameworks. Further research in larger and more diverse samples is warranted to consolidate these findings and to explore the role of MOLDES in clinical assessment and rehabilitation-oriented interventions.

## Availability of Data and Materials

The data that support the findings of this study are available on request from the corresponding author. The data are not publicly available due to privacy or ethical restrictions.
